# Effect of Exendin-4 on Autophagy Clearance in Beta Cell of Rats with Tacrolimus-induced Diabetes Mellitus

**DOI:** 10.1038/srep29921

**Published:** 2016-07-20

**Authors:** Sun Woo Lim, Long Jin, Jian Jin, Chul Woo Yang

**Affiliations:** 1 Transplant Research Center, Seoul St. Mary’s Hospital, College of Medicine, The Catholic University of Korea, Seoul, Korea; 2 Convergent Research Consortium for Immunologic Disease, Seoul St. Mary’s Hospital, College of Medicine, The Catholic University of Korea, Seoul, Korea; 3 Division of Nephrology, Department of Internal Medicine, Seoul St. Mary’s Hospital, College of Medicine, The Catholic University of Korea, Seoul, Korea

## Abstract

Growing evidence suggests that GLP-1 protects beta cells against various cellular injuries by modulating autophagy. In this study, we examined whether exendin-4 (Ex-4), a GLP-1 analog, had preventive effects on tacrolimus (Tac)-induced beta cell injury by improving autophagy clearance. Rats with Tac-induced diabetes mellitus exhibited increased autophagy-associated protein expression, light chain 3B levels, and autophagic vacuole numbers in pancreatic beta cells. Additionally, Tac increased autophagy in a dose- and time-dependent manner *in vitro*, and inhibition of autophagosome using 3-methyladenine reduced Tac-induced islet cell injury by decreasing reactive oxygen species production and apoptosis. Ex-4 treatment decreased Tac-induced hyperglycaemia, oxidative stress, and apoptosis, accompanied by decreased autophagy-associated protein expression and autophagosome numbers. *In vivo* and *in vitro* studies showed that Tac treatment impaired lysosomal function and autophagosome-lysosome fusion; these processes were improve by Ex-4 treatment. Moreover, addition of bafilomycin A1, an inhibitor of lysosomal function, abolished the protective effects of Ex-4. Our findings reveal that Tac-induced diabetes mellitus was a state of excessive burden of autophagosomes and impairment of autophagy clearance and that Ex-4 protected against Tac-induced pancreatic islet injury by reducing the burden of autophagosomes via activation of autophagosome clearance. Thus, Ex-4 had therapeutic effects on Tac-induced pancreatic beta cell injury.

Autophagy is a highly conserved, homeostatic intracellular process in eukaryotic cells and function to facilitate the degradation of misfolded proteins, removal of damaged or superfluous organelles, and resistance to invading intracellular microorganisms. Autophagy can also act as an adaptive mechanism that ensures cell survival during metabolic, genotoxic, or hypoxic stress conditions. However, extensive autophagy or inappropriate clearance of autophagy can result in cell death^1^,^2^.

Tacrolimus (Tac) is a widely used maintenance immunosuppressant in renal transplant recipients (KTRs). However, Tac causes considerable metabolic abnormalities. In particular, new-onset diabetes after transplantation (NODAT) occurs in 10–25% of patients receiving Tac^3^,^4^. This condition reduces graft survival and increases the risk of infectious and cardiovascular diseases. Moreover, Tac-induced NODAT is related to the direct toxic effects or oxidative stress of Tac on pancreatic beta cells.

Highly selective dipeptidyl peptidase IV (DPP IV) inhibitors control hyperglycaemia by stimulating insulin production via prevention of the degradation of two major incretins, glucagon-like peptide-1 (GLP-1) and glucose inhibitory peptide (GIP). In addition, DPP IV inhibitors have protective effects against inflammation, oxidative injury, and apoptotic cell death in various disease models^5^,^6^,^7^,^8^,^9^. Therefore, the use of DPP IV inhibitors may be ideal in patients with Tac-induced diabetes; however, it remains unclear whether the tissue-protective effects of DPP IV inhibitors are also effective in Tac-induced pancreatic islet cell injury.

The role of autophagy in Tac-induced pancreatic islet cell injury is still not clear. Therefore, in this study, we investigated whether exendin-4 (Ex-4), a long acting GLP-1 analog, affected Tac-induced pancreatic islet injury by modulation of autophagy. Using experimental rats and INS-1 cells, we studied the status of autophagy in Tac-induced pancreatic islet cell injury and the effects of Ex-4 on modulating Tac-induced autophagy. The results of our study clearly demonstrated the rationale for use of Ex-4 in the management of Tac-induced diabetes mellitus based on autophagy regulation.

## Results

### Tac-induced diabetes mellitus, oxidative stress, and apoptosis were suppressed by Ex-4 treatment

**Figure 1:**
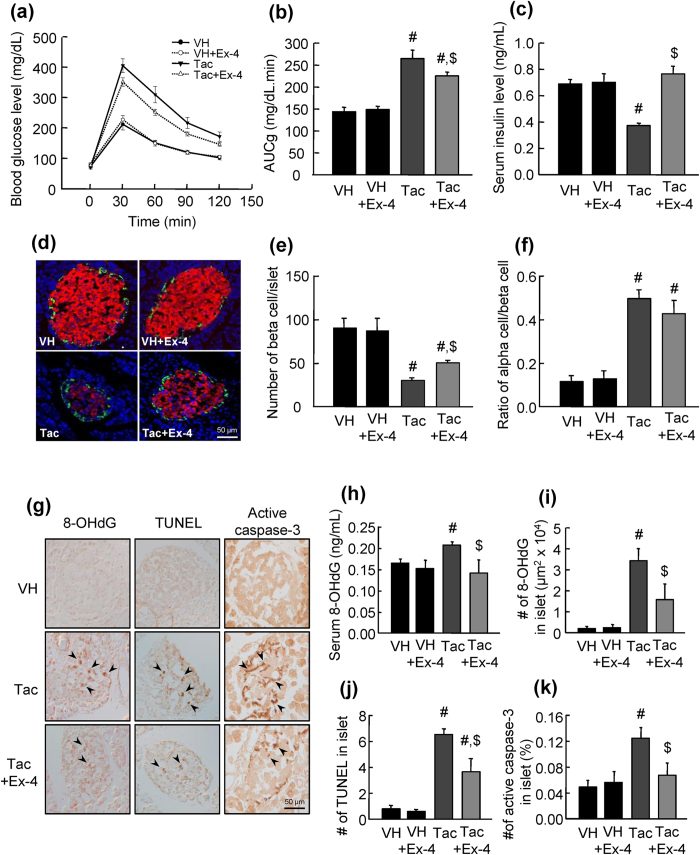
Tac-induced diabetes mellitus, oxidative stress, and apoptosis were reduced by Ex-4 treatment in rats. Blood glucose levels were measured using IPGTTs (**a**), calculation of the AUCg from IPGTTs (**b**), and serum insulin levels (**c**). (**d**) Representative insulin (red) and glucagon (green) staining of islets. (**e**) Number of beta cells per islet. (**f**) Ratio of the number of alpha cells to the number of beta cells. (**g**) Representative staining for 8-OHdG, TUNEL, and active caspase-3 in the experimental group. (**h**,**i**) Serum 8-OHdG levels and number of 8-OHdG-positive cells in islets. (**g**) Number of TUNEL-positive cells in islets. (**k**) Number of active caspase-3-positive cells in islets. Arrowheads indicate 8-OHdG-, TUNEL-, and active caspase-3-positive cells in islets. The data are presented as means ± standard errors (SEs). n = 8 per group. ^#^*P* < 0.05 versus the VH or VH plus Ex-4 groups; ^$^*P* < 0.05 versus the Tac group. Scale bar = 50 μm.

Rats treated with Tac exhibited symptoms of diabetes mellitus, as demonstrated by increased area under the curve of glucose (AUCg) values and lower levels of serum insulin as compared with those in rats treated with vehicle (VH). Concomitant treatment with Ex-4 reversed these changes (Fig. 1a–c and Table 1). Morphologically, islets treated with Tac showed lower immunoreactivity for insulin and decreased numbers of beta cells compared with those in rats treated with VH; these effects were reversed by Ex-4 (Fig. 1d,e). The number of alpha cells identified by the ratio of glucagon-positive cells to insulin-positive cells was markedly increased in the Tac group but not in the Tac plus Ex-4 group (Fig. 1f). Oxidative stress, as measured using 8-hydroxy-2′-deoxyguanosine (8-OHdG), was higher in pancreatic islet tissue sections and serum from rats in the Tac group than that from rats in the VH group; however, Ex-4 treatment attenuated these changes (Fig. 1g–i). Apoptosis, as measured using terminal deoxynucleotidyl transferase dUTP nick end labelling (TUNEL) assays and enumeration of active caspase-3-positive cells, was markedly increased in the Tac group as compared with those in the VH and VH plus Ex-4 groups; notably, however, Ex-4 treatment reduced both parameters (Fig. 1g,j,k).

### Ex-4 reduced Tac-induced autophagy substrate levels and autophagosome formation

**Figure 2:**
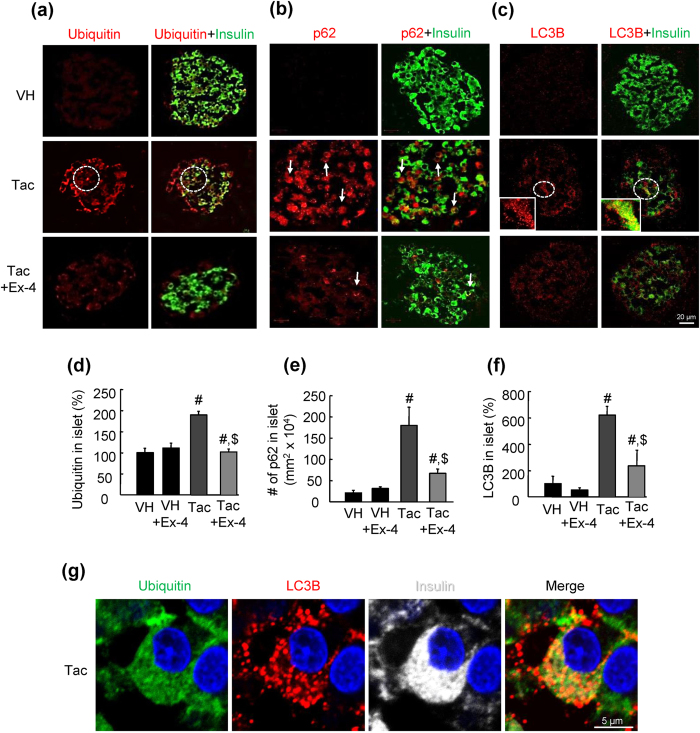
Ex-4 reduced Tac-induced autophagy substrate expression and autophagosome formation in rats. (**a–f**) Representative images of double immunofluorescence of insulin (green) and p62 (red), ubiquitin (red), or LC3B (red) and their quantificative analysis in islets from experimental rats. Scale bar = 20 μm. (**g**) High-power confocal image showing ubiquitin (green), LC3B (red), and insulin (white) in Tac-treated pancreatic islets. Arrows indicate cells positive for both p62 and insulin. Dotted circles indicate cells positive for insulin and ubiquitin or LC3B. Scale bar = 5 μm. The data are presented as means ± SEs. n = 8 per group. ^#^*P* < 0.05 versus the VH or VH plus Ex-4 groups; ^$^*P* < 0.05 versus the Tac group.

**Figure 3:**
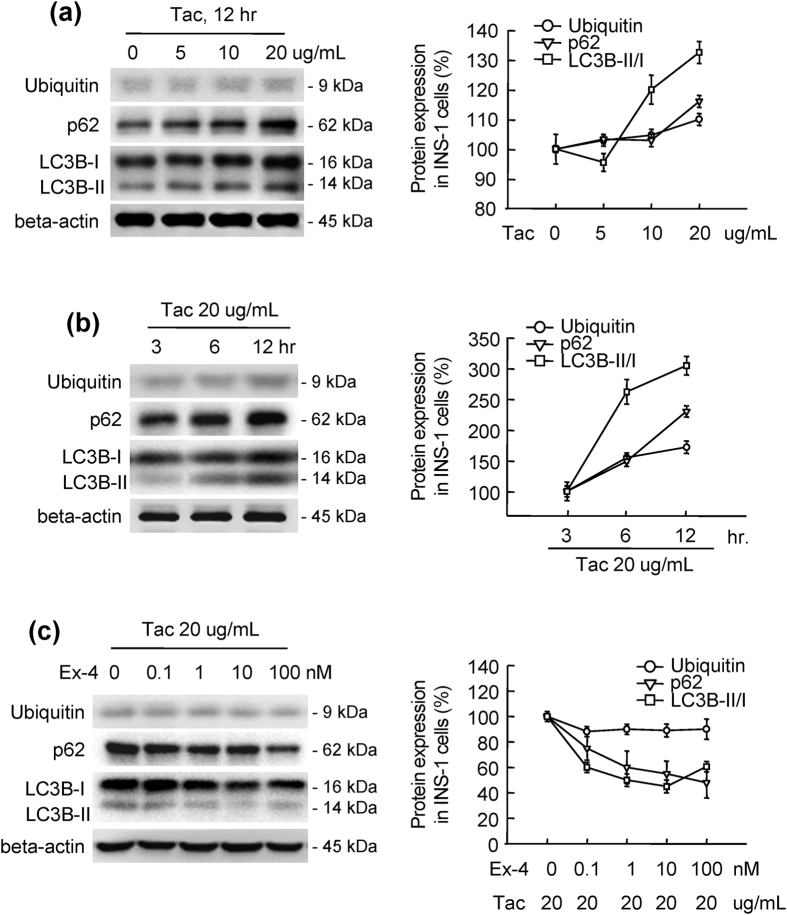
Ex-4 reduced Tac-induced autophagy substrate expression and autophagosome formation in INS-1 cells. (**a**–**c**) Representative immunoblots and quantification of the concentration- and time-dependent expression of p62, ubiquitin, and LC3B-I/LC3B-II (as a ratio). *In vitro* data are presented as means ± SEs of at least three independent experiments. Open circles, ubiquitin; open triangles, p62; open rectangles, LC3B-I/LC3B-II.

Next, we examined alterations in autophagy substrate (ubiquitin protein aggregate and p62) levels and autophagosome formation (as measured by the expression of microtubule-associated protein 1 light chain 3 beta [LC3B]) in tissue sections from experimental rats. In the Tac group, confocal microscopy revealed that increased numbers of ubiquitin-, p62-, and LC3B-positive cells coincided with insulin positivity, indicating the presence of these markers in beta cells (Fig. 2a–c). Moreover, the numbers of ubiquitin-, p62-, and LC3B-positive cells were significantly higher in the Tac group than in the VH group. However, Ex-4 attenuated these changes (Fig. 2d–f). High-magnification images revealed that ubiquitin was colocalized with LC3B in a punctate manner in beta cells from rats in the Tac group (Fig. 2g). Thus, to determine whether this effect was dependent on time or Tac concentration, we observed the expression of ubiquitin, p62, and LC3B in cultured INS-1 cells. Interestingly, the expression levels of these proteins were increased with Tac treatment in a time- and concentration-dependent manner (Fig. 3a,b). Addition of Ex-4 attenuated these increases in a concentration-dependent manner during Tac treatment (Fig. 3c).

### Autophagy was observed in pancreatic beta cells in rats with Tac-induced diabetes mellitus

**Figure 4:**
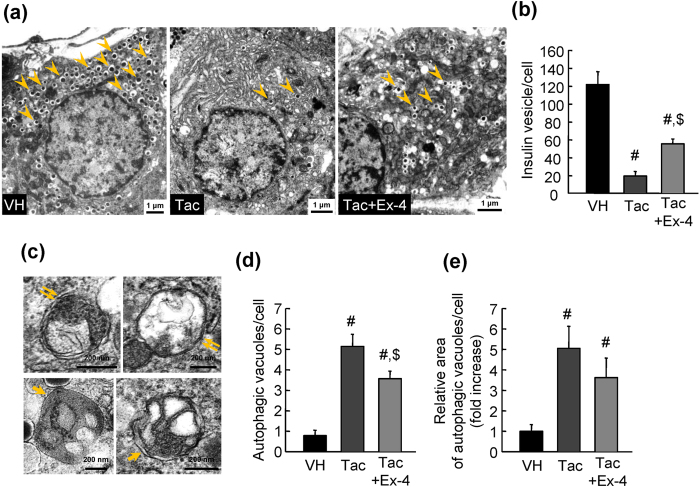
Electron microscopy of pancreatic beta cells in rats with Tac-induced diabetes mellitus. (**a**,**b**) Quantitative graphs demonstrating dispersion of insulin granules (arrowheads) in the cytoplasm cells in the VH, VH plus Ex-4, Tac, and Tac plus Ex-4 groups. Scale bar = 1 μm. (**c**) Higher-magnification images from the Tac-treated group, showing organelles surrounded by double or single membranes representing autophagosomes (double arrows) or autolysosomes (arrows), respectively. Scale bar = 200 nm. (**d** and **e**) Numbers and areas of autophagic vacuoles containing autophagosomes and autolysosomes in the VH, VH plus Ex-4, Tac, and Tac plus Ex-4 groups. The data are presented as means ± SEs. ^#^*P* < 0.05 versus the VH group; ^$^*P* < 0.05 versus the Tac group.

To visualise the induction of autophagy in experimental animals, we performed transmission electron microscopy (TEM) on islets (Fig. 4). Beta cells from Tac-treated rats contained markedly reduced numbers of insulin granules compared with those in the VH group (Fig. 4a,b). High-magnification TEM images (Fig. 4c) showed double membrane-bound vacuoles containing intact cytoplasmic organelles (suggestive of autophagosomes) and single membrane-bound vacuoles containing degraded cytoplasmic organelles (suggestive of autolysosomes). Counting and measuring the areas of autophagic vacuoles/vesicles in micrographs also indicated that there was a significant increase in autophagy in cells from the Tac group (Fig. 4d,e). Combined treatment with Ex-4 and Tac attenuated the formation of autophagic vacuoles and induced recovery of the number of insulin granules.

### Tac-induced pancreatic beta cell injury was associated with autophagy

**Figure 5:**
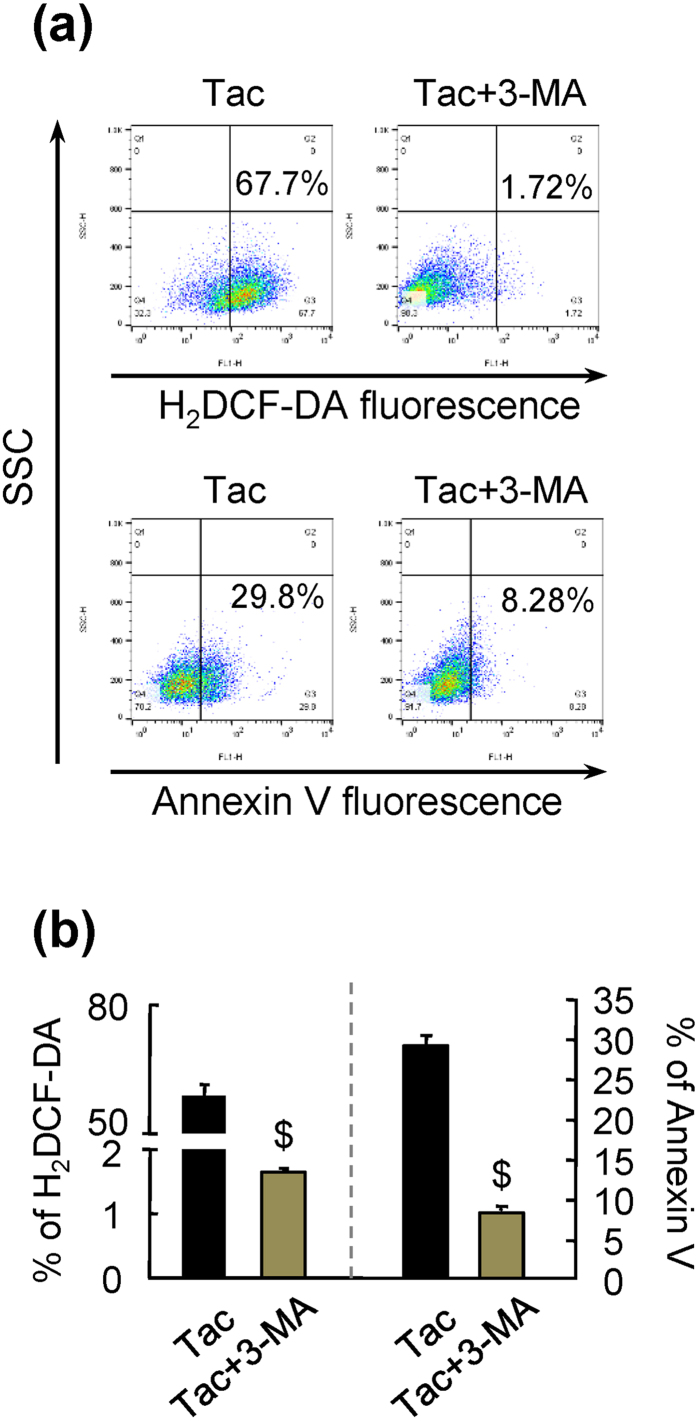
Effects of 3-MA on Tac-induced oxidative injury and apoptosis in INS-1 cells. Oxidative injury and apoptosis were evaluated by measuring H_2_DCF-DA and annexin V fluorescence using flow cytometry. (**a**,**b**) Effects of 3-methyadenine (3-MA), an inhibitor of autophagosome formation, on H_2_DCF-DA and annexin V fluorescence. *In vitro* data are presented as means ± SEs of at least three independent experiments. ^$^*P* < 0.05 versus the Tac group.

To determine whether Tac-induced beta cell injury was mediated through autophagy, we treated INS-1 cells with the autophagy inhibitor 3-methyladenine (3-MA). The effects of autophagy inhibition were evaluated by measuring changes in fluorescence levels of 2′,7′-dichlorodihydrofluorescein diacetate (H_2_DCF-DA, for detecting reactive oxygen species [ROS] production) and annexin V (for detecting apoptosis). 3-MA, which inhibits class III phosphatidylinositol 3-kinase to block autophagosome formation at the early stage of autophagy, significantly decreased the percentages of H_2_DCF-DA- and annexin V-positive Tac-treated INS-1 cells (Fig. 5). These data suggested that Tac-induced pancreatic beta cell injury resulted in a state of excessive autophagosome formation and that autophagy was responsible for Tac-induced beta cell injury.

### Ex-4 reversed Tac-induced impaired autophagy clearance by improving lysosomal function

**Figure 6:**
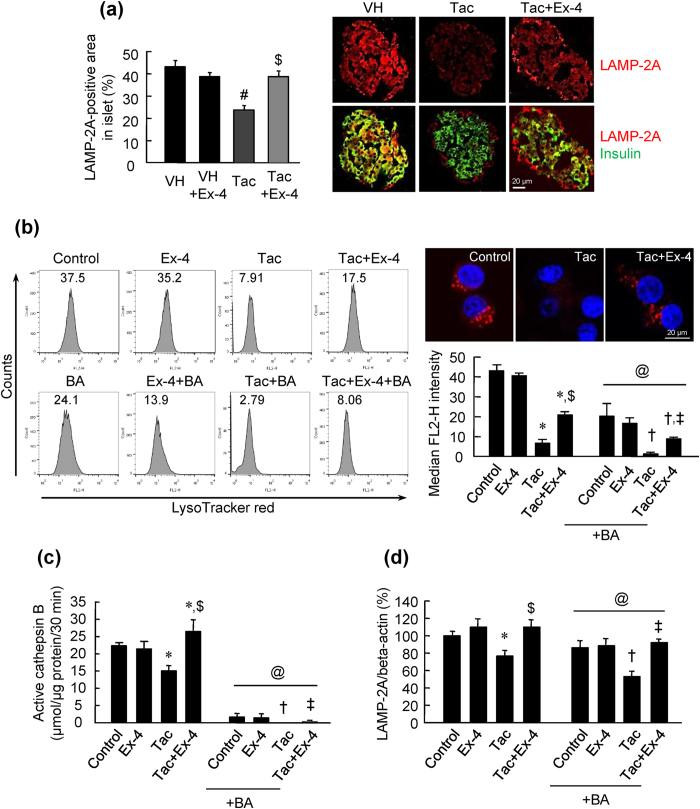
Ex-4 reversed Tac-induced impairment of autophagy clearance by improving lysosomal function. (**a**) Quantification and representative images of double immunofluorescence of LAMP-2A (red) and insulin (green) in islets of experimental rats. (**b**) Lysosomal acidification using LysoTracker red in INS-1 cells treated with Tac and Ex-4 in the presence or absence of bafilomycin A1 (BA). Fluorescence intensity was detected by flow cytometry and confocal images. (**c**) Active cathepsin B using fluorogenic assays in INS-1 cells. (**d**) Immunoblot analysis of LAMP-2A/beta-actin in INS-1 cells. Data are presented as means ± SEs of at least three independent experiments. ^#^*P* < 0.05 versus the VH, VH plus Ex-4, or control groups; ^$^*P* < 0.05 versus the Tac group; ^†^*P* < 0.05 versus the VH plus BA or Ex-4 plus BA groups; ^‡^*P* < 0.05 versus the Tac plus BA group; ^@^*P* < 0.05 versus the corresponding group. Scale bar = 20 μm.

Next, we evaluated lysosomal function to determine whether Tac-induced pancreatic islet injury was associated with autophagy clearance. First, we examined lysosomal protein expression in experimental animals. Tac treatment significantly decreased lysosomal-associated membrane protein 2A (LAMP-2A) expression within beta cells, as shown in Fig. 6a; however, treatment with Ex-4 attenuated these effects. Next, we measured the activation of lysosomal function using INS-1 cells under combined Tac plus Ex-4 treatment. Cells were stained with LysoTracker and analyzed using flow cytometry and confocal imaging (Fig. 6b). Tac-treatment significantly reduced the fluorescence intensity, whereas Ex-4 treatment recovered the fluorescence intensity to that of control cells. Similar patterns were observed for cathepsin B and LAMP-2A (Fig. 6c,d). Bafilomycin A1 (BA) was used as a negative control of lysosomal function and was shown to abolish lysosomal acidification.

### Ex-4 enhanced autophagosome-lysosome fusion in Tac-induced pancreatic beta cell injury

**Figure 7:**
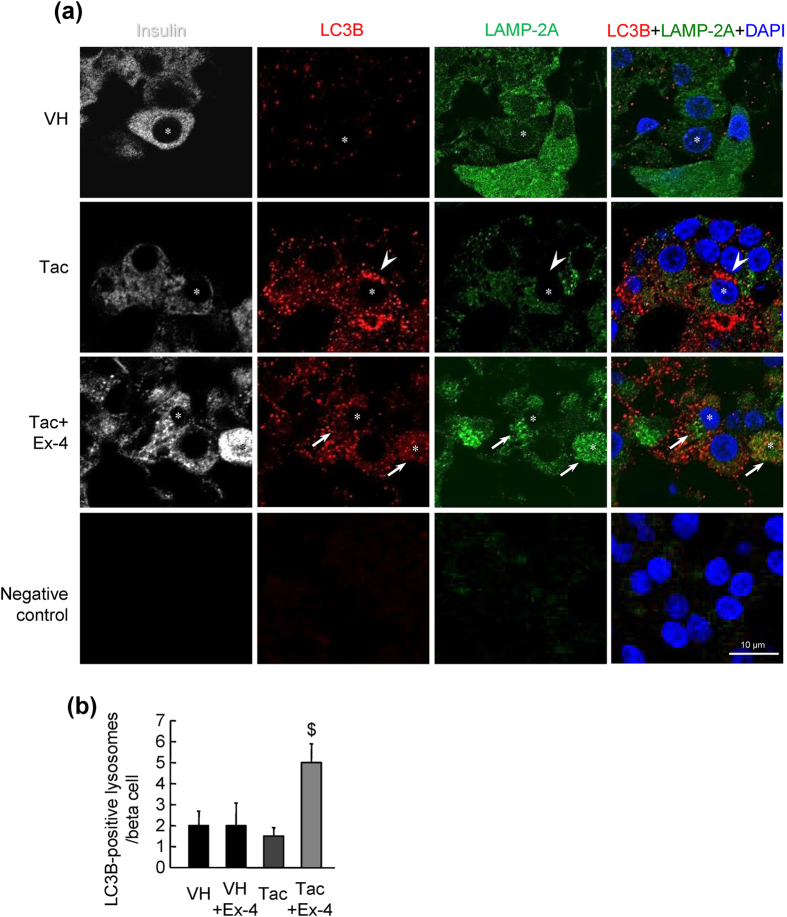
Ex-4 enhanced autophagosome-lysosome fusion in rats with Tac-induced diabetes mellitus. (**a**) Representative confocal immunofluorescence images of LC3B (autophagosome marker, red), LAMP-2A (lysosome marker, green), insulin (pancreatic beta cell marker, white) in islets from experimental groups. Increased LC3B punctate staining was not colocalized with LAMP-2A in the Tac group (arrowhead). Double-positive LC3B and LAMP-2A (arrows) cells were observed in the Tac plus Ex-4 group. Asterisks indicate insulin-positive cells. The negative control cells were treated without primary antibodies. (**b**) Numbers of LC3B-positive lysosomes in the Tac and Tac plus Ex-4 groups. Data are presented as means ± SEs of at least three independent experiments. ^$^*P* < 0.05 versus Tac group. Scale bar = 10 μm.

**Figure 8:**
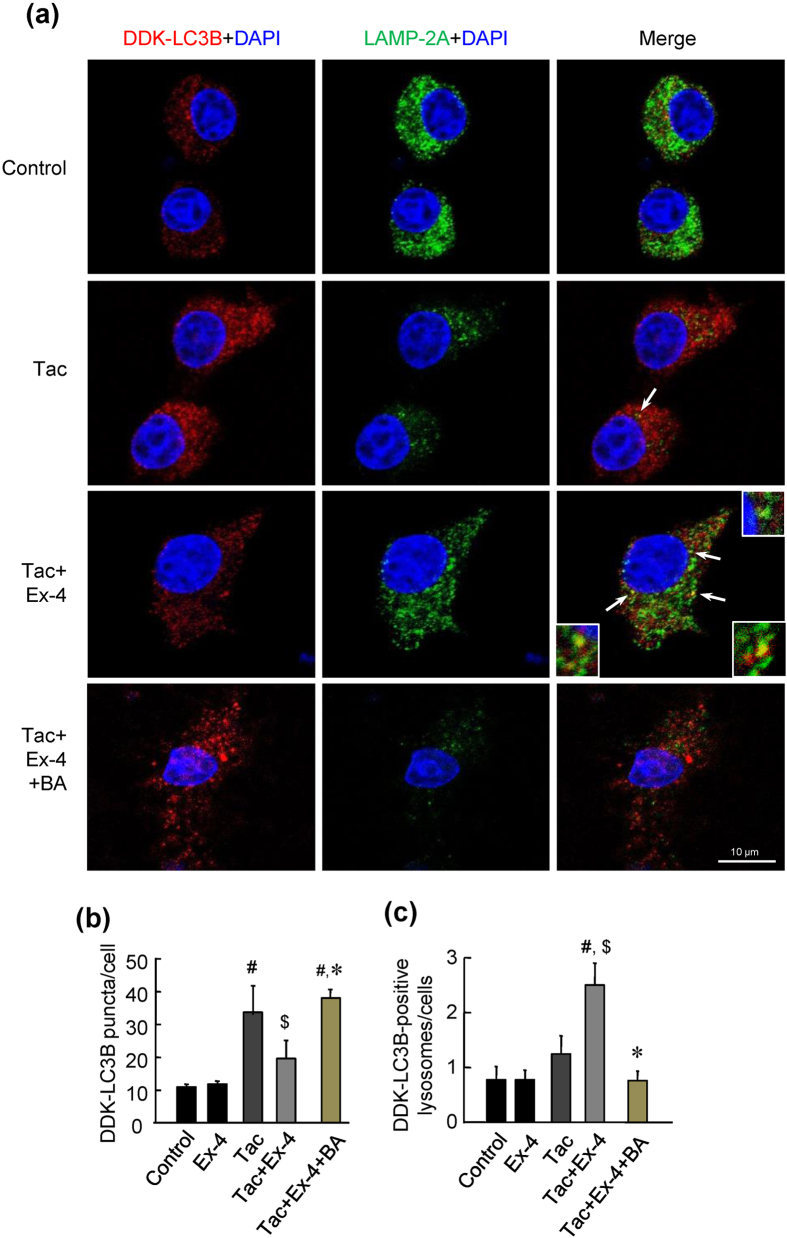
Ex-4 enhanced autophagosome-lysosome fusion in Tac-treated INS-1 cells. (**a**) Representative confocal immunofluorescence images of DDK-LC3B (autophagosome marker, red) and LAMP-2A (lysosome marker, green). DDK-LC3B was transfected into INS-1 cells, and cells were then treated with Tac and/or Ex-4. LC3B punctate staining was detected by immunostaining with anti-DDK antibodies. Arrows indicate double-positive fluorescence (yellow). Scale bar = 10 μm. (**b**) Quantitative analysis of LC3B puncta per cell. (**c**) Numbers of LC3B-positive lysosomes. Bafilomycin A1 (BA) was used as a negative control for autophagosome-lysosome fusion. Data are presented as means ± SEs of at least three independent experiments. ^#^*P* < 0.05 versus the control and Ex-4 groups; ^$^*P* < 0.05 versus the Tac group; *P < 0.05 versus the Tac plus Ex-4 group.

To examine autophagosome-lysosome fusion in pancreatic beta cells, we examined the colocalization of LC3B (an autophagosome marker), LAMP-2A (a lysosome marker), and insulin (a pancreatic beta cell marker) in tissue sections from experimental rats. Punctate LC3B expression in beta cells was obviously observed in the Tac group, and the combined treatment with Ex-4 attenuated this change. In contrast, LAMP-2A expression was decreased in the Tac group compared with that in the VH group, but was recovered in the Tac plus Ex-4 group (Fig. 7a). Moreover, combined treatment with Ex-4 markedly enhanced LC3B localization to lysosomes (Fig. 7b). Next, we treated INS-1 cells with Tac and/or Ex-4 after transfection with DDK-tagged LC3B and then performed double immunofluorescence to detect DDK and LAMP-2A. Similar to the results in experimental rats, Ex-4 reduced LC3B punctate expression and enhanced colocalization of LC3B and LAMP-2A as compared with that in the Tac group (Fig. 8a–c). We used BA as a negative control for autophagosome-lysosome fusion. Addition of BA markedly induced accumulation of LC3B and suppressed the expression of LAMP2A.

### The protective effects of Ex-4 were causally related to lysosomal function

**Figure 9:**
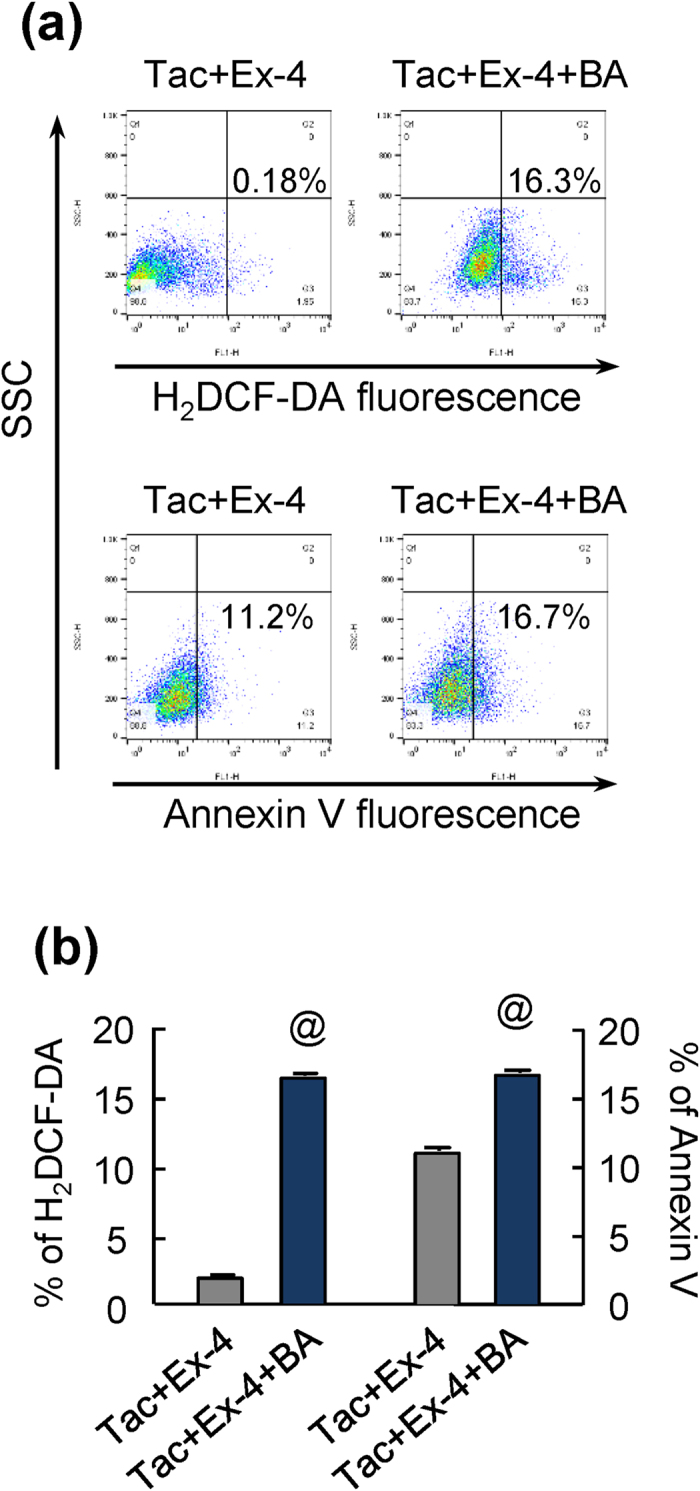
Effects of BA on oxidative injury and apoptosis in INS-1 cells in combination with Tac and Ex-4 treatment. (**a**) Oxidative injury and apoptosis were evaluated by measuring H_2_DCF-DA and annexin V fluorescence levels using flow cytometry. (**b**) Effects of bafilomycin A1 (BA), a specific inhibitor of the vacuolar type H^ + ^-ATPase in lysosomes, on the percentages of H_2_DCF-DA- and annexin V-positive cells. *In vitro* data are presented as means ± SEs of at least three independent experiments. ^@^*P* < 0.05 versus the Tac plus Ex-4 group.

To determine whether the protective effects of Ex-4 on Tac-induced injury were mediated through improved lysosomal function, INS-1 cells were treated with BA, and the effects of BA were evaluated by measuring changes in H_2_DCF-DA and annexin V. Inhibition of lysosomes by BA in Tac plus Ex-4 in INS-1 cells significantly increased the percentages of H_2_DCF-DA- and annexin V-positive cells as compared with that in Tac plus Ex-4 cells not treated with BA (Fig. 9).

## Discussion

In this study, we investigated the effects of Ex-4 on autophagy in the context of Tac-induced pancreatic beta cell injury. The results of our study clearly demonstrated that Tac treatment increased autophagy substrate expression and autophagosome formation. However, addition of Ex-4 reduced these alterations by improving lysosomal dysfunction and the frequency of autolysosome-lysosome fusion. These findings provide a rationale for use of Ex-4 in the management of Tac-induced diabetes mellitus in clinical practice.

To date, few studies have examined autophagy in dysfunctional beta cells of rats with Tac-induced diabetes mellitus. Therefore, in this study, we examined markers of autophagy substrates and autophagosomes in rats with Tac-induced diabetes mellitus. In this study, we used p62 and ubiquitinated protein aggregates as markers of autophagy substrates based on previous reports showing that such toxic aggregates are increased in neurodegenerative diseases, such Huntington’s disease, and in pancreatic beta cell-specific autophagy-deficient mice^10^,^11^. Pancreatic beta cells are known to bear a high burden of protein synthesis and folding. Therefore, the disposal of misfolded or denatured proteins is important, and the autophagosome/lysosome system plays a key role in this process^12^. Here, we found that Tac treatment increased the levels of p62 and ubiquitin-positive beta cells and LC3B. Furthermore, TEM observations revealed abundant autophagic vacuoles accompanying reduced numbers of insulin-bearing vesicles. These findings suggested that Tac-induced diabetes mellitus was characterised by overloaded protein aggregates and the accumulation of large amounts of autophagic vacuoles.

In addition, to clarify the casual relationship between Tac-induced beta cell injury and autophagy, we evaluated the effects of an autophagy inhibitor in cultured INS-1 cells (Fig. 5). Our findings showed that blocking autophagosome formation by 3-MA significantly decreased oxidative stress and apoptosis caused by Tac, suggesting that excessive autophagosome formation was responsible for Tac-induced oxidative stress and apoptotic cell death.

Next, we evaluated autophagy clearance by measuring lysosomal function because lysosomes are the main organelles involved in processing autophagosomes. From our study, we found that Tac treatment impaired lysosomal function, as demonstrated by the increased lysosomal pH and reduced hydrolase enzyme activity. Subsequently, damaged lysosomes could not fuse with autophagosomes, as demonstrated by reduced colocalization of markers for lysosomes and autophagosomes. Combined treatment with Ex-4 significantly reversed Tac-induced lysosomal defects, e.g., increased LAMP-2A and cathepsin B activity, reduced lysosomal pH, and decreased the autophagosome-lysosome fusion rate. Thus, we suggest that lysosomal impairment may be involved in Tac-induced pancreatic beta cell injury and that Ex-4 may improve the clearance of Tac-induced autophagosomes by enhancing lysosomal activity.

In this study, we also found that BA treatment increased oxidative stress and apoptotic cell death as compared with that in the Tac plus Ex-4 group without BA treatment, suggesting that the protective effects of Ex-4 on Tac-induced pancreatic beta cells were closely associated with lysosomal function. Thus, we cautiously concluded that Tac-induced pancreatic beta cell injury may be causally associated with lysosomal dysfunction. However, we found that Ex-4 had protective effects, even in the presence of BA. Ex-4 was still able to “rescue” the phenotype of Tac treatment, including maintaining activity of cathepsin B and LAMP-2A. The reason for this protective effect of Ex-4 in the state of lysosomal inhibition was unclear. However, it is possible that Ex-4 may act on a step of the autophagy process well before lysosomal acidification and may also affect lysosomal acidification. Alternatively, Ex-4 may exert protective effects via direct antioxidative and anti-apoptotic effects or via an AMP-dependent protein kinase (AMPK)-dependent mechanism, which could affect beta cell survival^13^,^14^.

The role of GLP-1 analogs in autophagy is unclear. Liraglutide (LRG), another GLP-1 analog, inhibits free fatty acid (FFA)-induced cell injury in hepatocytes or pancreatic beta cells by activating autophagy^15^,^16^. LRG has also been shown to improve cardiac function in mice fed a high-fat diet (HFD) by increasing autophagy^17^. On the other hand, consistent with our results, LRG has been shown to reduce LC3 levels, autophagosome formation, and apoptosis following chronic exposure of renal tubular epithelial cells to high glucose (HG)^18^. These contradictory findings suggest that GLP-1 analogs may act differently on autophagy according to the intensity and duration of stress rather than different mechanisms of action. FFA and HFD are relatively weak stresses compared with Tac or HG. Therefore, activation of autophagy is required to prevent FFA- or HFD-induced cell injury. However, Tac and HG induce a state of excessive autophagosome formation, and reduced autophagy is needed to prevent the cell injury. In this situation, GLP-1 analogs may exert their protective effects by activating autophagy or clearing autophagosomes according to the intensity and duration of stress.

**Figure 10:**
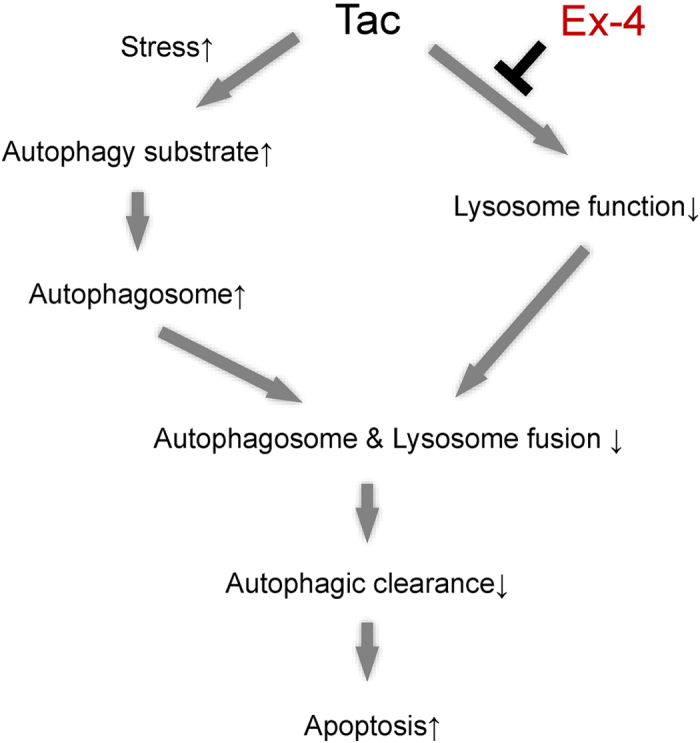
Proposed mechanism through which Ex-4 protects against Tac-induced diabetes mellitus, involving autophagy clearance from pancreatic beta cells. Tac treatment augments autophagosome formation in pancreatic beta cells to adapt to stress and lysosomal dysfunction. However, autophagosomes are not effectively degraded due to impaired autophagy clearance (lysosomal dysfunction and autophagosome fusion with lysosomes). Therefore, excessive autophagosomes lead to apoptotic cell death. In this process, Ex-4 improves the autophagic clearance rate by enhancing lysosomal function and autophagosome fusion with lysosomes.

The effects of Ex-4 on autophagy in Tac-induced pancreatic beta cell injury are summarized in Fig. 10. Tac treatment augments autophagosome formation in pancreatic beta cells to adapt to stress. However, autophagosomes are not effectively degraded due to impaired autophagy clearance (lysosomal dysfunction and autophagosome fusion with lysosomes). Therefore, excessive autophagosome formation leads to apoptotic cell death. In this process, Ex-4 improves autophagic clearance rate by enhancing lysosomal function and autophagosome fusion with lysosomes. Through this mechanism, Ex-4 may protect against Tac-induced pancreatic beta cell dysfunction.

In conclusion, impaired lysosome-associated autophagic degradation or clearance is an important element in the pathogenesis of Tac-induced pancreatic beta cell injury, and Ex-4 may be useful for management of Tac-induced diabetes mellitus by modulating autophagy.

## Materials and Methods

### Animal experiments

Eight-week-old male Sprague Dawley rats (Charles River Technology, Seoul, Korea) were housed in a controlled-light environment at the animal facility of the Catholic University of Korea. The Animal Care and Use Committee of the Catholic University of Korea approved the experimental protocol (CUMC-2012-0117-04), and all procedures performed in this study were in accordance with ethical guidelines for animal studies. Animals were maintained on a 12/12-h light/dark cycle, with a 0.05% salt diet (Research Diets, New Brunswick, NJ, USA) and water ad libitum. After acclimatization for 1 week, weight-matched rats were randomised to four groups (n = 8 each) and were treated daily with a subcutaneous (s.c.) injection of 1.5 mg/kg Tac (Prograft; Astellas Pharma, Ibaraki, Japan) or 1 mL/kg VH (olive oil; Sigma-Aldrich, St. Louis, MO, USA) with or without Ex-4 (1 μg/kg, intraperitoneal [i.p.] injection; Sigma-Aldrich) for 4 weeks. Routes of administration and doses of drugs were chosen based on previous studies^19^,^20^. Rats were pair-fed, and their body weights were monitored daily. After the 4-week treatment, animals were housed individually in metabolic cages (Tecniplast, Buguggiate, Italy) for the measurement of urine volume over 24 h. The following day, animals were anaesthetised, and blood samples and tissue specimens were obtained for further analysis. Serum Scr levels, blood urea nitrogen (BUN) levels, and trough levels of Tac in the blood were measured as described previously^20^.

### Pancreatic function and islet size

An intraperitoneal glucose tolerance test (IPGTT) was performed at the end of the 4 weeks of treatment. Briefly, after 1 day of fasting, 50% dextrose (1.5 g/kg) was injected, and the blood glucose concentration was measured just before and at 30, 60, 90, and 120 min after the injection using a glucose analyser (Accu-Check; Roche Diagnostics, Basel, Switzerland). The AUCg was calculated by trapezoidal estimation from the values obtained in the IPGTT. The fasted serum insulin level was measured using a sandwich enzyme-linked immunosorbent assay (ELISA; Millipore Corp., St. Charles, MO, USA). For the quantification of islet size, the numbers of beta cells or alpha cells were assessed in each using quantification of captured images from immunohistochemistry with insulin or glucagon (TDI Scope Eye Version 3.6 for Windows; Seoul, Korea). Insulin- or glucagon-positive cells were evaluated by counting approximately 20 randomly selected nonoverlapping islets for the eight animals in each group.

### Cell culture

The rat insulinoma cell line INS-1 was grown in RPMI-1640 medium supplemented with 10 nM HEPES, 1 mM sodium pyruvate, 2 mM L-glutamine, 50 mM 2-mercaptoethanol (all from Sigma-Aldrich), 100 IU/mL penicillin, 100 mg/mL streptomycin, and 10% foetal bovine serum (all from Wisent Bio, Saint-Bruno, QC, Canada) in a humidified atmosphere containing 5% CO_2_. Cells were seeded in culture plates and treated with Tac (5–20 μg/mL) and Ex-4 (0.1–100 nM) with or without 3-MA (10 mM; Sigma-Aldrich) and BA (5 nM; Sigma-Aldrich) for 3–12 h.

### Cell transfection

INS-1 cells were transfected with DDK-LC3B plasmids (RR209172; OriGene Technologies, Rockville, MD, USA) using Lipofectamine 2000 (Invitrogen, Carlsbad, CA, USA) according to the manufacturers’ instructions. After the cells had been immunostained with anti-DDK and anti-LAMP-2A antibodies following treatment with Tac (20 μg/mL), Ex-4 (10 nM), and BA (5 nM) for 12 h, the localization of LC3B and LAMP-2A was observed using a Zeiss LSM700 confocal microscope (Carl Zeiss MicroImaging GmbH, Jena, Germany).

### Antibodies

Primary antibodies used for confocal microscopy or immunoblot analysis were as follows: anti-insulin (I2018, Sigma-Aldrich; 18-0067, Invitrogen, Camarillo, CA, USA), anti-glucagon (8233; Cell Signaling Technology, Danvers, MA, USA), anti-active caspase-3 (AB3623; Millipore Corp.), anti-8-OHdG (MOG-020P; JaICA, Shizuoka, Japan), anti-p62 (AB56416, Abcam, Cambridge, UK; GP62-C, Progen Biotechnik GmbH, Heidelberg, Germany), anti-ubiquitin (PA5-17067; Thermo Fisher Scientific, Rockford, IL, USA), anti-LC3B (L7543, Sigma-Aldrich; AB168831, Abcam; ALX-803-080, ENZO Life Sciences, Inc., Farmingdale, NY, USA), anti-LAMP-2A (3900-100; BioVision Inc., Milpitas, CA, USA), and anti-DDK (TA50011-100, OriGene Technologies).

### Preservation of tissues and double immunofluorescence

Pancreas tissues were preserved by *in vivo* perfusion through the abdominal aorta. The anaesthetised animals were first perfused with phosphate-buffered saline (PBS) to flush blood from the tissues. Dissected pancreas tissues were immersed in periodate-lysine-2% paraformaldehyde solution at 4 °C overnight. After being rinsed in PBS, tissues were dehydrated in a graded series of ethanol and embedded in polyester wax (polyethylene glycol 400 distearate; Polysciences, Warrington, PA, USA). Sections (5 μm thick) were cut and mounted on gelatin-coated glass slides for further analysis. Colocalization was confirmed using double immunofluorescence on the sections. The dewaxed sections were incubated with retrieval solution (pH 6.0) and 0.5% Triton X-100 (Sigma-Aldrich) and then washed with PBS. Nonspecific binding sites were blocked with a mixture of 10% normal donkey serum (Jackson ImmunoResearch, West Grove, PA, USA) and 0.5% bovine albumin serum (Sigma-Aldrich). Sections were incubated overnight at 4 °C in a mix of primary antibodies. The next day, sections were incubated with secondary antibodies conjugated with Alexa Fluor 488 (Molecular Probes, Invitrogen Life Sciences, Carlsbad, CA, USA), Cyanine3 (Cy3; Jackson ImmunoResearch), or Alexa Fluor 680 (Molecular Probes) for 2 h at room temperature. Subsequently, the tissue sections were stained with 4′,6-diamidino-2-phenylindole (DAPI; Vector Laboratories, Burlingame, CA, USA) for nucleic acid staining. Stained tissues were viewed using a Zeiss LSM700 confocal microscope (Carl Zeiss MicroImaging GmbH).

### Immunostaining for active caspase-3 and 8-OHdG

The dewaxed sections were incubated with retrieval solution (pH 6.0), methanolic H_2_O_2_, and 0.5% Triton X-100 and then washed with PBS. Nonspecific binding sites were blocked with a mixture of 10% normal donkey serum (Jackson ImmunoResearch). Sections were incubated overnight at 4 °C with anti-active caspase-3 or anti-8-OHdG antibodies. The next day, sections were incubated with peroxidase-conjugated secondary antibodies for 2 h at room temperature. For detection of peroxidase, 3,3′-diaminobenzidine (DAB; Vector Laboratories) was used as a chromogen to produce a brown colour. The quantification was performed in approximately 20 randomly selected nonoverlapping islets per animal in each group. Quantitative analysis was performed by calculating the percent positive area showing the same intensity per islet using histogram equalization (TDI Scope Eye).

### Immunoblot analysis

INS-1 cells were plated in 96-well plates at a density of 25,000 cells/well in complete RPMI medium. After 24 h, the cells were treated with serum-free RPMI medium containing Tac (5–20 μg/mL) and Ex-4 (0.1–100 nM) with or without BA. At the indicated times, INS-1 cells were lysed in 10 mM Tris (pH 7.5) containing 1% sodium dodecyl sulphate (SDS) and 1 mM NaVO_4_. Equal amounts of proteins were resolved using SDS-polyacrylamide gel electrophoresis and electroblotted onto polyvinylidene fluoride membranes (Millipore Corp.). The membranes were incubated for 1 h at room temperature in blocking solution containing 5% nonfat milk (BD Difco, Sparks, MD, USA) in PBS, followed by overnight incubation at 4 °C with the primary antibodies diluted in Signal Boost immunoreaction enhancer (Millipore Corp.). The next day, the blots were incubated for 1 h with peroxidase-conjugated secondary antibodies (Cell Signaling Technologies). Antibody binding was detected with commercial chemiluminescence kits (ATTO Corp., Tokyo, Japan). Quantification was performed using relative densities, with the control group set at 100%; densities were normalised to that of the beta-actin bands from the same gel (Quantity One version 4.4.0; Bio-Rad, Hercules, CA, USA).

### TEM

After fixation in 2.5% glutaraldehyde in 0.1 M phosphate buffer, pancreatic tissues were postfixed with 1% O_S_O_4_ and embedded in Epon 812. Ultrathin sections were cut, stained with uranyl acetate/lead citrate, and photographed with a JEM-1200EX transmission electron microscope (JEOL Ltd., Tokyo, Japan). Sections were scanned randomly at 20 different spots per sample at 5000× magnification. The numbers and areas of autophagic vacuoles (autophagosomes and autolysosomes) per cell in the scanned areas were measured using imaging software (TDI Scope Eye).

### LysoTracker staining

One day after cell seeding, the cells were treated with serum-free RPMI medium containing Tac (20 μg/mL) and Ex-4 (10 nM) in the presence or absence of BA (5 nM). After 12 h, the cells were incubated with 100 nM LysoTracker Red DND-99 (Molecular Probes) for 1 h at 37 °C according to the manufacturer’s instructions and then analyzed using a FACSCalibur flow cytometer (BD Biosciences, San Jose, CA, USA) or confocal microscope (Carl Zeiss Microscopy GmbH).

### Cathepsin B activity

Cathepsin B activity was evaluated using a fluorogenic substrate, Z-Arg-Arg AMC, according to the manufacturer’s protocol (Millipore Corp.). Released free AMC was detected fluorometrically at an excitation wavelength of 360 nm and an emission wavelength of 460 nm using a VersaMax Microplate Reader (Molecular Devices, Sunnyvale, CA, USA). The results were normalised to the protein concentration.

### TUNEL assay

The TUNEL assay was performed according to the manufacturer’s instructions using an ApopTag Plus Peroxidase *In Situ* Apoptosis Detection kit (Millipore Corp.) using tissue sections. The number of TUNEL-positive cells was counted in approximately 20 randomly selected nonoverlapping islets per animal in each group.

### Detection of 8-OHdG

Oxidative DNA damage was evaluated based on the level of 8-OHdG in serum using a competitive ELISA (Cell Biolabs, San Diego, CA, USA).

### Apoptosis

One day after cell seeding, the cells were treated with serum-free RPMI medium containing Tac (20 μg/mL) and Ex-4 (10 nM) in the presence or absence of 3-MA (10 mM) or BA (5 nM) for 12 h. Trypsinised INS-1 cells were treated with 5 μL of fluorescein isothiocyanate (FITC)-conjugated annexin V (BD Biosciences) in 1× binding buffer (BD Biosciences) for 15 min at room temperature according to the manufacturer’s protocol. The stained cells were analyzed using flow cytometry on a FACSCalibur instrument (BD Biosciences). Values are expressed as the percentage of fluorescent cells relative to the total cell count.

### Intracellular ROS generation

One day after cell seeding, the cells were treated with serum-free RPMI medium containing Tac (20 μg/mL) and Ex-4 (10 nM) in the presence or absence of 3-MA (10 mM) or BA (5 nM). After 12 h, cells were incubated with 10 μM H_2_DCF-DA (Molecular Probes) for 1 h at 37 °C according the manufacturer’s instructions and then analyzed using a FACSCalibur (BD Biosciences). Values are expressed as the percentage of fluorescent cells relative to the total cell count.

### Statistical analysis

The data are expressed as the mean ± standard error (SE) of at least three independent experiments. Multiple comparisons between groups were performed by one-way analysis of variance (ANOVA) with Bonferroni post-hoc tests using IBM SPSS Statistics (Version 19.0; IBM Corp., Armonk, NY, USA). Results with *P* values of less than 0.05 were considered significant.

## Additional Information

**How to cite this article**: Lim, S. W. *et al*. Effect of Exendin-4 on Autophagy Clearance in Beta Cell of Rats with Tacrolimus-induced Diabetes Mellitus. *Sci. Rep.* **6**, 29921; doi: 10.1038/srep29921 (2016).

## Figures and Tables

**Table 1:** Effect of Ex-4 on basic parameters.

	VH	VH + Ex-4	Tac	Tac + Ex-4
Body weight (g)	359 ± 14	364 ± 7	348 ± 4	339 ± 10
Urine volume (mL)	13 ± 1	10 ± 1	58 ± 6^1,2^	39 ± 5^1,2,3^
Scr (mg/dL)	0.31 ± 0.03	0.33 ± 0.01	0.49 ± 0.01^1,2^	0.43 ± 0.02^1,2,3^
BUN (mg/dL)	18.1 ± 0.4	15.2 ± 0.3	56.2 ± 7.3^1,2^	43.8 ± 1.6^1,2,3^
Blood Tac level (ng/mL)	—	—	9.8 ± 0.6	10.8 ± 0.5

VH, vehicle; Ex-4, Exendin-4; Tac, tacrolimus; Scr, serum creatinine; BUN, blood urea nitrogen. Values are S.E. (*n* = 8). ^1^P < 0.05 vs. VH, ^2^P < 0.05 vs. VH + Ex-4, ^3^P < 0.05 vs. Tac.
